# The effects of charismatic leadership on performance: exploring key boundary conditions at multilevel

**DOI:** 10.3389/fpsyg.2025.1487886

**Published:** 2025-04-15

**Authors:** Linyuan Zhang, Jee Young Seong, Doo-Seung Hong, Hye Jung Yoon

**Affiliations:** ^1^School of Society and Law, Shandong Women's University, Jinan, Shandong, China; ^2^College of Business and Economics, Jeonbuk National University, Jeonju, Republic of Korea; ^3^Department of Sociology, Seoul National University, Seoul, Republic of Korea; ^4^Department of Business Administration, Sejong University, Seoul, Republic of Korea

**Keywords:** charismatic leadership (CL), high-performance work system (HPWS), group performance orientation (GPO), creativity, performance, boundary condition

## Abstract

**Introduction:**

Charismatic leadership (CL) plays an indispensable role in facilitating organizational change, stimulating creativity, and attaining performance objectives. Given the growing attention on the relationship between CL, creativity and performance, we establish a multilevel model that examines the moderated mediation effect of CL on task performance through creativity. This model considers both individual and group levels while accounting for contextual factors such as high-performance work systems (HPWS) at the individual level and group performance orientation (GPO) at the group level.

**Methods:**

Our analysis of field data gathered from members of work groups offers robust empirical support for our multilevel framework. Data were collected from team members and their leaders in Korean private-sector organizations, utilizing a final sample of 109 teams consisting of 1,080 members and 109 leaders for our analysis. Conducted ordinary least square (OLS) regression analysis were used to test the hypotheses.

**Results:**

The findings of this study indicate that CL exhibits higher creativity at the individual level when HPWS is low compared to high (*compensatory effect*). In contrast, CL produces higher group creativity at the group level when GPO is high rather than low (*additive effect*). Additionally, individual-level creativity mediates the relationship between the interaction of CL and HPWS at the individual level and task performance. The influence of the interactive effect of CL and GPO on performance at the group level is mediated by group-level creativity.

**Conclusion:**

This study emphasizes the significance of adopting a multilevel interactive perspective on creativity and performance while providing evidence for how leadership and its boundary conditions affect creativity and performance.

## 1 Introduction

Charismatic leadership (CL) is a type of leadership that influences the behavior of subordinates through their charm and magnetism (House, 1977). It is crucial for driving organizational change, fostering creativity, and achieving performance objectives. The relationship between CL and creativity and performance has garnered significant attention from scholars (Bass, 1985; McClelland and Boyatzis, 1982). However, there are still substantial gaps and deficiencies in understanding how CL affects creativity and performance (Zhang and Xia, 2011), particularly regarding the multilevel perspective within different contextual settings (i.e., “situational opportunities or constraints that affect the occurrence and meaning of organizational behavior as well as functional relationships between variables,” Johns, 2006, p. 386).

In contemporary research, investigating individual-level factors can provide more immediate insights into employees’ attitudes, behaviors, and active engagement in the workplace (Seong et al., 2023). Conger and Kanungo (1988) observed that charismatic leaders are uncomfortable with maintaining the status quo and tend to take decisive actions to change. Charismatic leaders, being sensitive to environmental changes and exhibiting non-conformist behavior (Conger and Kanungo, 1988), can influence subordinates to emulate their personal risk-taking and unconventional behaviors in line with social learning theory (Bandura, 1977). By fostering employees’ adoption of risk- and change-related attitudes and behaviors through leadership actions, the confidence and psychological safety necessary for navigating creative risks are enhanced (Guzzo et al., 1993), thereby promoting individual creativity. Consequently, task performance is also impacted (Oldham and Cumming, 1996; Cheng and Osman, 2021; Vila-Vázquez et al., 2024). However, individuals within organizations are exposed to various management practices beyond just leadership styles (Chiang et al., 2015). Scholars in human resource management have proposed that exploring the interactive effects of these management practices is crucial for understanding how organizations influence group member behavior (Becker and Gerhart, 1996; Delery and Doty, 1996). From this perspective emerges the notion that individuals experience interconnected employee management practices encompassing selection processes, training programs, performance appraisals, compensation schemes, and job design arrangements. This study examines how the interaction between this set of management practices, high-performance work systems (HPWS) (Gong et al., 2010; Jiang et al., 2012; Takeuchi et al., 2007; Yang et al., 2020), and CL influences task performance through individual creativity.

Given that many organizations regard groups as their fundamental structure (Pieterse et al., 2012), and individual behaviors often manifest and execute within this context (Shalley et al., 2004), effectively managing creativity and performance necessitates the identification of both group members’ creativity potential and goal achievement capabilities, as well as a comprehensive understanding of group dynamics.

This challenges research and practice with an inherent multilevel focus, requiring insights into individual-level processes and group-level dynamics. To analyze creativity more comprehensively, scholars have recently recognized and embraced the significance of goal orientation theory (Gong et al., 2009; Hirst et al., 2009; Janssen and van Yperen, 2004). Hence, to better comprehend the inconsistent findings from previous studies on multilevel research (Chang et al., 2014; Jiang et al., 2012; Liu et al., 2017) and address the gaps resulting from predominantly examining single-level constructs (Chiang et al., 2015), this study attempts to probe the interactive effect of group-level CL and GPO (Gully and Phillips, 2005; Hirst et al., 2009) on group creativity, and thus on group performance.

This research seeks to advance the literature on leadership, creativity, and performance by developing a multilevel moderated mediation framework proposing that CL, in conjunction with its boundary conditions, such as HR systems and group climate, is used for performance via creativity at the individual and group levels. Our analysis also contributes to the growing interest in multilevel influences on organizational behavior and dynamics, providing additional evidence and implications for the potential of this approach.

## 2 Hypothesis development

The role of a group leader encompasses establishing short- and long-term objectives, providing guidance to the group, and exerting influence within the group (Guzzo and Dickson, 1996). Previous research has demonstrated a significant association between leadership style and creativity and performance outcomes (Juyumaya and Torres, 2023; Wolfgang and Andrea, 2018). Among them, especially CL (the degree of a manager’s effect on followers in the leader-follower relationship; Seong and Hong, 2018) has been found to affect both creative thinking and behavior and overall performance (Dong et al., 2010; Wang and Yuan, 2016).

Despite some progress in research on the relationship between leadership and creativity, there are still inconsistencies and gaps in understanding (Zhang and Xia, 2011). Scholars suggest that more diverse perspectives should be explored to enrich this study area (Shalley et al., 2004). Recent developments highlight the importance of contextual or situational conditions, which are defined as “implicit or explicit cues provided by external entities regarding the desirability of potential behaviors” (Meyer et al., 2010, p. 122). Contextual conditions play a prominent role in predicting human behavior over extended periods (Cooper and Withey, 2009; Meyer et al., 2010) by exerting psychological stress on individuals within groups to encourage desired behaviors and hinder unwanted ones.

Creativity in the workplace has been defined as generating novel and improved ideas regarding organizational processes and outcomes to enhance practices and performance (Amabile, 1996; Anderson et al., 2014; Shalley et al., 2004). Individual creativity refers to generating new and valuable ideas by a group member (Gong et al., 2013). Research on creativity typically explores how individual variables interact with contextual factors to inspire creative thinking (Shalley et al., 2004; Woodman et al., 1993; Seong and Choi, 2023). Given that individuals are influenced by multiple management practices simultaneously, we propose that HPWS, which is defined as an integrated set of human resource management practices (HRMPs) such as selection processes and disciplinary management aimed at achieving superior performance and competitive advantage (Arokiasamy et al., 2024; Chiang et al., 2015; Ren et al., 2023), moderates the relationship between CL and individual-level creativity.

At the individual level, the interaction between CL and HPWS may suppress creativity. Amabile (1988) discussed a classic three-component model of individual-focused creativity from a social-psychological perspective, which includes motivation, domain-relevant skill/expertise, and creative thinking process. These components are closely associated with different dimensions of HPWS. For instance, when organizations engage in rigorous employee recruitment processes to find individuals who align with their group’s qualities (Epitropaki and Martin, 2005), it can lead to a high degree of homogeneity among group members. Simultaneously, this selection process fosters strong identification among group members towards each other. Drawing upon social identity (Tajfel, 1982) and social categorization (Turner, 1987) theories, employees perceiving high identity and high congruence tend to form tightly knit groups where conformity is favored over disrupting the status quo to maintain membership. In such a tightly knit environment, individuals are apt to avoid risk rather than challenge existing relationships to receive social approval while avoiding criticism and negative judgments from others. Those people may hinder knowledge exchange relevant to the domain-specific skills/expertise and inhibit the creative thinking process. Furthermore, strict disciplinary management practices and effective rules/regulations reinforce non-challenging employee behaviors by domesticating them within organizational boundaries.

In cases involving high-HPWS environments, as discussed by Zhou (2003) based on Bandura’s social cognitive theory (Bandura, 1991, 1986), leaders hold positions of authority that exert dominant influence over their employees’ behavior and shape both the content and direction of their work outcomes resulting in tense interactions that impact employee psyche. When informed about the higher risk associated with accomplishing creative work, this category of employees experiences a reduction in their beliefs regarding job success. In such instances, even when leaders extend support and offer incentives for creative endeavors, these employees may perceive such supportive behavior as burdensome or even confrontational. The more supportive actions leaders exhibit towards these employees, the more likely they will view these behaviors as impositions imposed upon them, consequently leading to heightened resistance psychology. Thus, belief and motivation for achieving creative outcomes are further impeded.

By contrast, the positive potential of individual CL toward individual creativity is more likely to be realized in a low level of HPWS, where individuals are more likely to be part of a heterogeneous work unit and are not strongly constrained by normative stress to conform. Employees are willing to underscore their uniqueness “to feel idiosyncratic and different from other team members in their thoughts, feelings, and behaviors” (Janssen and Huang, 2008; p. 72). Consistent with job demands-resources (JD-R; Demerouti et al., 2001) and discrepancy-arousal theory (Capella and Greene, 1982), when there is a discrepancy between work requirements and individual resources, as well as personal needs and supplies from the work setting (e.g., a low level of HPWS), group members are motivated to seek approaches and tactics that fulfill both job demands and their own needs. As such, this can facilitate motivation for changing the status quo, communication, and exchange of domain-relevant skills/expertise and the creative thinking process. In an environment with a low level of HPWS, which may reduce concerns about disrupting existing membership dynamics and interpersonal harmony resulting from rules and arrangements, individuals are more inclined to take social risks and deviate from established routines and rules (Ashmore et al., 2004). Therefore, we propose the following hypothesis:

Hypothesis 1: HPWS will negatively moderate the relationship between individual-level CL and individual creativity, such that the relationship between individual-level CL and individual creativity will be less positive when HPWS is high than it is low.

Group creativity is defined as the generation of new and valuable ideas by group members working together (Shin and Zhou, 2007). At the group level, we propose a contrasting moderating effect for GPO compared to the hypothesized effect at the individual-level HPWS. GPO reflects group goal preferences in achievement-oriented contexts that influence group actions and reactions and a cognitive-motivational focus on a group’s collective abilities and capacities (Dweck, 1986; Dweck and Leggett, 1988; Elliot and Church, 1997). An externally oriented motivation characterizes it and influences group information exchange and sharing behaviors (Gong et al., 2013).

Charismatic leaders present a vision or strategies that offer solutions for complex challenges and difficult miscellaneous diseases faced by groups or organizations, attracting followers who trust in their exceptional abilities to manage critical issues (Bligh and Kohles, 2010). When there is a high level of GPO, it becomes more feasible to reach a consensus on cognitive-motivation frameworks or shared mental models concerning what matters to the group and how to accomplish it (Mathieu et al., 2000). This facilitates group actions such as developing shared goals and expectations, which can enhance the psychological safety climate. Unlike individual-level HPWS, which may lead individuals to seek social approval and worry about potential negative evaluations, a high level of group-level GPO fosters a psychologically safe work environment and increases trust derived from CL (Meglino and Ravlin, 1998; Smith-Jentsch et al., 2005). This heartens open communication, collaboration, information exchange, and knowledge sharing (Meglino and Ravlin, 1998; Smith-Jentsch et al., 2005). Knowledge and information are strategic resources for groups as they contribute to gaining heterogeneous competitive advantages and enhancing creative capabilities. The exchange and sharing of knowledge and information provide opportunities for knowledge integration, strengthen the mutual transformation of explicit and tacit knowledge, and boost employee communication. Accordingly, this stimulates new ideas, methods, and creative motivation (Hong et al., 2006; Jackson et al., 2006). In other words, since shared knowledge and information are essential sources of creative ideas and approaches within groups or organizations, the likelihood of creative generation depends on adequate knowledge exchange and sharing.

Furthermore, in groups with high GPO, the positive emotions derived from high CL, such as trust, psychological safety, and optimism about the future, may be amplified. According to broaden-and-build theory (Fredrickson, 2001), positive emotions expand an individual’s cognitive and behavioral repertoire by broadening their scope of thinking and action (Fredrickson, 1998). When individuals experience positive emotions in non-threatening situations, tend to engage in non-specific actions while becoming more focused and open-minded. In this state of openness, individuals are inclined to explore new approaches, develop innovative problem-solving strategies, and embark on original endeavors. These novel ideas, experiences, and actions significantly enhance individual cognition and behavior (Strumpfer, 2006), further expanding group behavior.

Consequently, group creative behavior is likely to improve. Positive emotions can also facilitate the development of enduring psychological and social resources through a constructive process based on “expansion” (Fredrickson and Losada, 2005). This constructive function is crucial in providing resources for fostering creative behavior. Therefore, we propose the following hypothesis:

Hypothesis 2: GPO will positively moderate the relationship between group-level CL and group creativity such that the relationship between group-level CL and group creativity will be more positive when GPO is high than when it is low.

Dong et al. (2010), DeGroot et al. (2000), and Bass (1985) have demonstrated that CL fosters high motivation for tasks and articulates goals, thereby improving task motivation and elaboration. This ultimately leads to superior performance outcomes; a higher level of CL can promote both individual task performance and group performance. Charismatic leaders set up quickly comprehensible goals for their subordinates while ensuring subordinates identify and align with their ideals and ambitions (McClelland and Boyatzis, 1982). They subsequently inspire group members to exert diligent efforts towards shared goals and visions by providing guidance, support, courage, and confidence. While pursuing these common objectives and visions, they cultivate mutual trust among group members while stimulating the group to improve individual task performance and overall group performance (McClelland and Boyatzis, 1982).

In addition, charismatic leaders with a heightened sensitivity to environmental changes do not rest on their laurels (Conger and Kanungo, 1988; Meslec et al., 2020). Their behavior is characterized by adaptability, flexibility, and enterprising and adventurous spirits, enabling them to implement decisive measures that challenge the status quo swiftly (Conger and Kanungo, 1988), fostering creative behaviors. Creativity is the cornerstone for pursuing corporate objectives and ensuring profitability (Scott, 1995), while contributing to organizational growth and competitive development (Baer and Oldham, 2006).

Creativity is the cornerstone for pursuing corporate goals and ultimately securing profits (Scott, 1995) while contributing to organizational growth and competitive development (Baer and Oldham, 2006). Frymire (2006) argued that for organizations to thrive in the global economy, they must cultivate creative employees capable of applying creativity across all aspects of their work and their entire units. Asserted by predecessors (Oldham and Cumming, 1996; Scott et al., 2004), creativity can expedite work efficiency, enhance performance, and be conducive to organizational change and success.

Based on social cognitive theory (Bandura, 1991, 1986), a leader’s role modeling and support of subordinates increase subordinates’ self- and collective efficacy. When employees recognize their leaders’ support and trust, they develop greater confidence in themselves and their group, believing in their collective ability to overcome challenges. As a result, they are more likely to choose jobs that satisfy their and their collective abilities while being challenging. Group members focus on positively analyzing and resolving work-related difficulties and challenges, leading to improved creativity and, ultimately, improved performance.

By integrating these pathways along with contextual conditions and drawing upon prior research findings, this paper constructs a moderated mediation framework that explores the relationships between multilevel interaction of CL with boundary conditions and performance outcomes mediated by individual- and group-level creativity. Specifically, this study addresses a research model exploring the moderating effects of HPWS and GPO in the relationship between CL and task/group performance through individual- and group-level creativity. Therefore, we propose the following hypotheses:

Hypothesis 3: The interaction of individual-level CL and HPWS will affect task performance through individual creativity. Individual-level CL will have a less positive indirect relationship with task performance when HPWS is high than when it is low.

Hypothesis 4: The interaction of group-level CL and GPO will affect group performance through group creativity. Group-level CL will have a positive indirect relationship with group performance when GPO is high and a negative indirect relationship with group performance when GPO is low.

## 3 Method

### 3.1 Procedure and participants

For this research, we use the data collected from team members and their leaders in Korean private-sector firms (Company A and B, respectively) in different industries. At company A and B, the survey was conducted in two stages. In the first stage, team members were asked to complete the questionnaires available on the website. In the second stage, team leaders answered two sets of questionnaires, one to evaluate their team as a whole and the other to evaluate individual team members’ creativity and performance. After excluding missing values in the variables, the final sample size in company A was 933 employees (response rate = 73.6%). At company B, we collected data from all team members, except those who work independently with minimal interaction with their colleagues. After removing unreliable or unmatched responses, the final sample included 109 teams consisting of 1,080 members and 109 leaders.

### 3.2 Measures

The variables, excluding control variables, were measured using a seven-point Likert-type scale ranging from 1 (strongly disagree) to 7 (strongly agree).

### 3.3 Individual level data

#### 3.3.1 Charismatic leadership (CL)

This study adopted four items from the Multifactor Leadership Questionnaire suggested by Bass (1985) to assess CL, which has been widely employed in leadership research (Lowe et al., 1996; Waldman et al., 2001). The items are “My team leader makes people feel good to be around him/her,” “My team leader has complete confidence in him/her,” “My team leader communicates high performance expectations,” and “My team leader generates respect.” Cronbach’s alpha was 0.96.

#### 3.3.2 High-performance work system (HPWS)

Following Yang et al. (2020), this study adopted the Delery and Doty’s (1996) method to conceptualize and measure HR practices. The different dimensions of HR practices included career development, profit sharing, staffing, and performance-oriented appraisal etc. While there is no clear agreement on which specific HPWS practices should be prioritized, previous studies have presented several theoretical and methodological suggestions for why a systems approach, which considers HPWS as an integrated set of practices, is preferred in HPWS research (Becker and Huselid, 1998; Delery, 1998; Huselid and Becker, 1996). In particular, this paper adopts the concept of a unitary index (Way, 2002). The unitary index is generated by adding standardized scores for equally weighted components of differential HPWS based on the evaluation from team members.

#### 3.3.3 Individual creativity

Team leaders measured individual member’s creativity using Zhou and George’s (2001) 4-item scale. The items are as follows: “This member comes up with creative solutions to problems,” “This member comes up with new and practical ideas to improve team performance,” “This member suggests new ways to increase performance quality,” and “This member exhibits creativity on the job when given the opportunity to.”

#### 3.3.4 Task performance

Team leaders assessed individual member’s task performance with a three-item scale adapted to the context of Williams and Anderson (1991).

#### 3.3.5 Control variables

Age, gender, and education were used as control variables in the analyses because they influence the relationship between CL and individual-level outcomes (Bernerth and Aguinis, 2016). The controls include age (in years), gender (male = 1, female = 2), and education. Education was coded from 1 (“high school”) to 4 (“graduate school).

### 3.4 Group level data

#### 3.4.1 Charismatic leadership

Four items were prepared to assess the team members’ perception of charismatic leadership using Waldman et al. (2001). Sample items are: “Our team leaders have our complete confidence in them” and “Our team leader generates respect.”

#### 3.4.2 Group performance orientation (GPO)

Team members measured GPO of their team adapted from Bettencourt (2004).

#### 3.4.3 Team creativity

Team leaders also evaluated the creativity of their team by using four items from Zhou and George (2001).

#### 3.4.4 Team performance

Team leaders assessed the performance of their team using a 4-item scale reflecting group goal achievement adapted from Kearney and Gebert (2009) and Gonzalez-Mulé et al. (2016).

### 3.5 Analytic strategies

**Figure 1:**
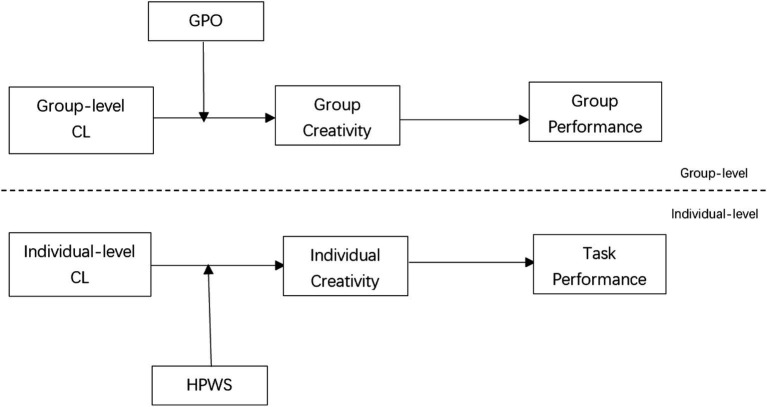
Hypothesis model. *N* = 933 (individual level); *N* = 109 (group level). CL, charismatic leadership; GPO, group performance orientation; HPWS, high performance work system.

Given that key variables were measured by different sources (i.e., team members assessed charismatic leadership, while team leaders evaluated creativity and task/team performance), we performed a regression analysis (Figure 1).

## 4 Results

First, we conducted a confirmatory factor analysis (CFA) to examine the distinctiveness of our scales: (1) CL, HPWS, individual creativity, and task performance at the individual level (Company A), and (2) CL, GPO, team creativity, and team performance at the team level (Company B) by using AMOS 23.0. We compared our original four-factor model with plausible alternative models. The results demonstrated that the expected four-factor model at the individual level provides a significantly better fit than the alternative models (χ^2^ (*df* = 234) = 617.15, *p* < 0.001, comparative fit index = 0.98, Tucker–Lewis Index (TLI) = 0.98, RMSEA = 0.04, SRMR = 0.02). As well, the expected four-factor model at the team level provides a significantly better fit than the alternative models (χ^2^ (*df* = 84) = 153.60, *p* < 0.001, comparative fit index = 0.95, Tucker–Lewis Index (TLI) = 0.94, RMSEA = 0.08, SRMR = 0.05).

Table 1 presents the study variables’ means, standard deviations, and correlations.

**Table 1:** Descriptive statistics, reliabilities, and relationships among individual-level and team-level variables.^a.^

Variables	Mean	SD	1	2	3	4	5	6	7	8	9
1. Age	37.11	9.34	–	–	–	–	–	–	–	–	–
2. Gender	1.42	0.49	−0.55^**^	–	–	–	–	–	–	–	–
3. Education	2.84	0.65	0.10^**^	−0.20^**^	–	–	–	–	–	–	–
4. Team size	11.41	7.91	–	–	–	–	0.36^**^	0.03	−0.13	−0.05	0.02
5. Team tenure	3.51	2.21	–	–	–		–	0.11	−0.16	0.08	0.19
6. CL	5.33, 5.31	1.27, 0.61	0.04	−0.13^**^	−0.00	−0.11	−0.13	*(0.96, 0.95)*	0.28^**^	0.18	0.22^*^
7. HPWS/GPO	4.77, 4.62	1.00, 0.45	−0.07^*^	−0.09^**^	0.00	−0.11	−0.13	0.45^**^	*(0.94, 0.80)*	0.21^*^	0.31^**^
8. Ind. Creativity/Team Creativity	5.21, 5.64	1.12, 0.69	0.00	−0.02	0.04	0.04	−0.02	0.11^**^	−0.01	*(0.97, 0.96)*	0.61^**^
9. Ind.performance/Team performance	4.61, 6.21	0.62, 0.55	0.04	−0.03	0.00	−0.05	−0.02	0.04	−0.04	0.53^**^	*(0.75, 0.85)*

^**a**^*N* = 933 individuals. *N* = 109 teams. Relationships among individual (team)-level variables are below (above) the diagonal. Alpha coefficient for reliability is listed in the diagonal across individuals and across teams. ^*^p < 0.05 (two-tailed test); ^**^p < 0.01 (two-tailed test).

### 4.1 Hypothesis testing

**Figure 2:**
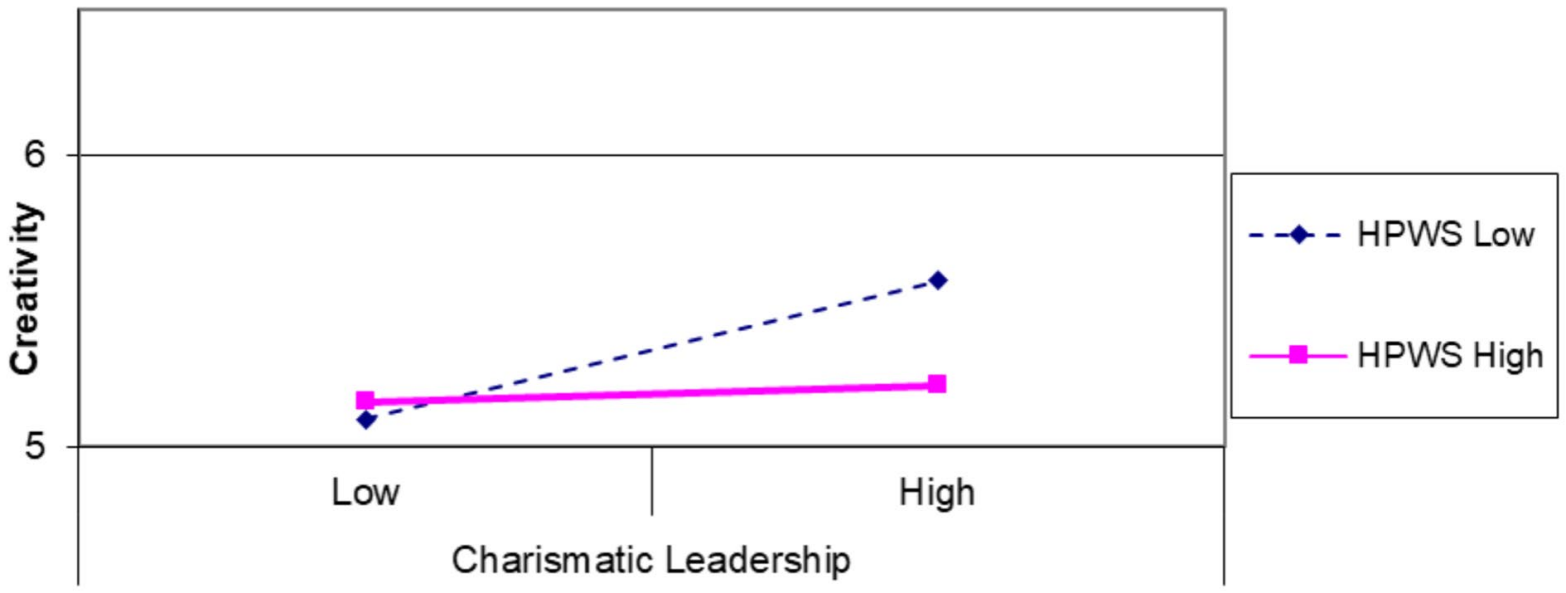
The interactive effect of charismatic leadership and HPWS on individual creativity (compensatory effect of leadership and HPWS system).

To test the hypothesis, we conducted ordinary least square (OLS) regression analysis. Hypothesis 1 states that HPWS will restrain the relationship between individual-level CL and individual creativity (Table 2). To investigate this significant interaction further, we conducted a simple slope analysis following Aiken and West’s (1991) approach. The interaction pattern depicted in Figure 2 aligns with our theoretical arguments. As anticipated, the effect of individual-level CL on individual creativity was less positive under High levels of HPWS compared to low levels, thus supporting hypothesis 1.

**Table 2:** Results of regression analyses of moderation at individual level^a^.

		Individual creativity		
	Variable	Model 1	Model 2	Model 3
Step 1	Age	−0.00	−0.02	−0.02
	Gender	−0.01	−0.00	−0.01
	Education	0.04	0.04	0.04
Step 2	CL		0.14^**^	0.55^**^
	HPWS		−0.08^*^	0.33^*^
Step 3	CL × HPWS			0.69^**^
	*Overall F*	0.62	3.44^**^	4.41^***^
	*R^2^*	0.00	0.02	0.03
	*F change*	0.62	7.64^**^	9.11^***^
	*R^2^ change*	0.00	0.02	0.01

*N* = 933. CL, charismatic leadership; HPWS, high-performance work system. ^a^Standardized regression coefficients are reported. ^*^p < 0.05, ^**^p < 0.01, ^***^p < 0.001.

**Figure 3:**
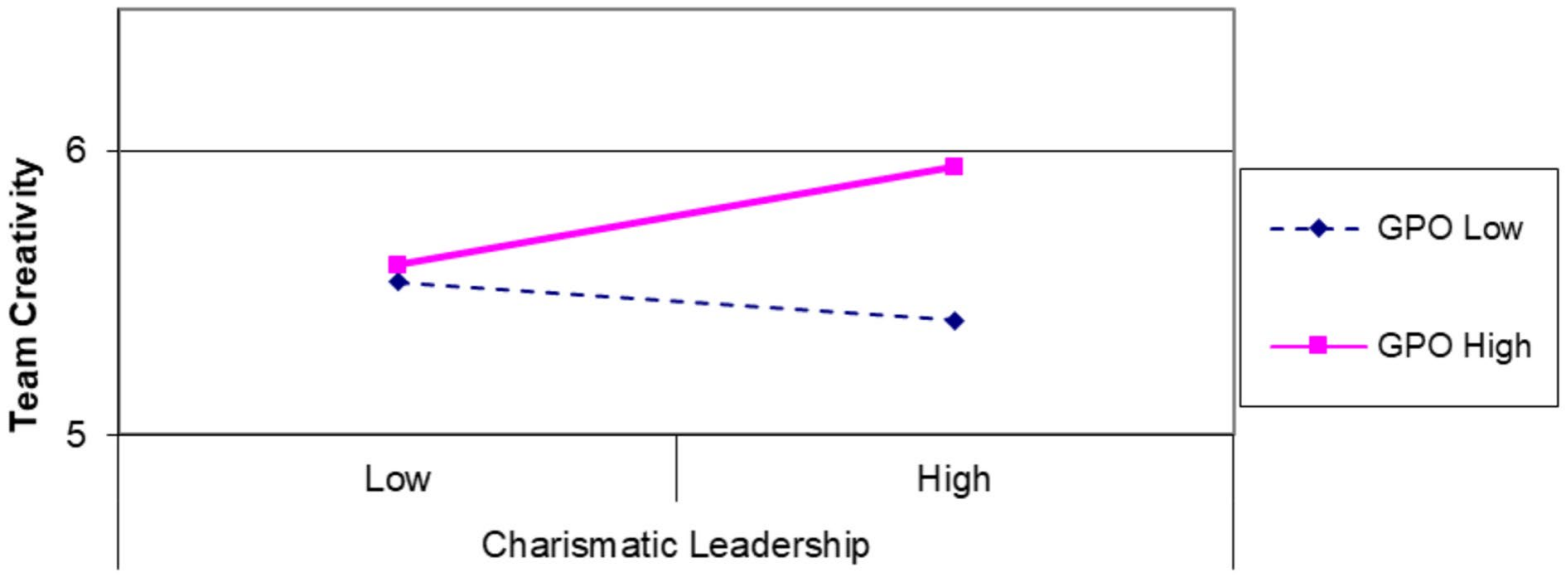
The interactive effect of charismatic leadership and group performance orientation on team creativity (Additive effect of leadership and performance-oriented climate).

Hypothesis 2 proposes that GPO will positively moderate the effect of group-level CL on group creativity (Table 3). Utilizing Aiken and West’s (1991) simple slope analysis, we graphed the effects of group-level CL on group creativity at high and low levels (±1 SD) of GPO, as shown in Figure 3. Consistent with our expectations, Figure 3 illustrated a significant enhancement in group creativity when group-level CL and GPO were high relative to other conditions. Thus, Hypothesis 2 received empirical support.

**Table 3:** Results of regression analyses of moderation at team-level.

		Team creativity		
	Variable	Model 1	Model 2	Model 3
Step 1	Team size	−0.10	−0.09	−0.09
	Team tenure	0.12	0.14	0.11
Step 2	CL		0.10	0.09
	GPO		0.23^*^	0.22^*^
Step 3	CL × GPO		−0.05	0.20^*^
	*Overall F*	0.82	2.51^*^	2.94^*^
	*R^2^*	0.02	0.09	0.13
	*F change*	0.82	4.14^*^	4.36^*^
	*R^2^ change*	0.02	0.07^*^	0.04^*^

*N* = 109. ^a^Standardized regression coefficients are reported. ^*^p < 0.05.

We then tested the conditional indirect effects of CL through creativity on task/ team performance at different levels of HPWS and GPO (Hypotheses 3 and 4). We used a bootstrapping procedure to probe the indirect effect at different moderator variable levels, such as HPWS and GPO. Following Preacher et al. (2007), we set high and low levels of HPWS at one standard deviation above and below the mean score of each HPWS. As shown in Table 4, the indirect effect was significant. As expected, the indirect effect of CL on task performance via OCB was conditional upon the level of PG DA fit. The indirect effect was significant and stronger at a low level of HPWS (*b* = 0.0529, bias-corrected bootstrap 95% CI [0.0302, 0.0762], excluding zero), while it was insignificant at a high level of HPWS. Thus, Hypothesis 3 was supported.

**Table 4:** Conditional indirect effect(s) of charismatic leadership on task performance at values of HPWS.

Path	Moderator	Effect	Boot SE	Boot LLCI	Boot ULCI
Simple path for low HPWS	3.8667	0.0529	0.0115	0.0302	0.0762
Simple path for high HPWS	5.9040	0.0040	0.0143	−0.0284	0.0316

95% bias-correlated CI. HPWS, high performance work system.

We expected that the indirect effect of CL on team performance through team creativity would become more strongly positive as the degree of GPO moved from a lower to a higher value. We also tested this indirect effect at GPO values equal to the mean plus and minus one standard deviation (Table 5). As expected, the indirect effect of CL on team performance via team creativity was conditional upon the level of GPO. The indirect effect was significant and stronger at a high level of GPO (b = 0.0805, bias-corrected bootstrap 95% CI [0.0200, 0.3400], excluding zero), while it was insignificant at a low level of GPO. Thus, Hypothesis 4 was supported.

**Table 5:** Conditional indirect effect(s) of charismatic leadership on team performance at values of GPO.

Path	Moderator	Effect	Boot SE	Boot LLCI	Boot ULCI
Simple path for low GPO	4.2198	−0.0394	0.0820	−0.2225	0.0969
Simple path for high GPO	5.0477	0.1614	0.0805	0.0200	0.3400

95% bias-correlated CI. GPO, group performance orientation.

## 5 Discussion

We designed this study to advance further research on management practices regarding the multilevel dynamics of the relationships between leadership, creativity, and performance within the contextual conditions of the HR system and group climate. The findings revealed distinct patterns of boundary conditions at the individual and group levels. Figures 2, 3 demonstrate that the association between CL and creativity is contingent upon the degree of HPWS and GPO at individual and group levels. Specifically, our results indicated that individual-level CL with a low level of HPWS would inspire higher individual creativity, consequently leading to improved task performance. For the group level, an interactive effect was observed between higher CL and higher GPO, positively influencing group creativity and, thus, overall group performance. Conversely, when combined with low CL and GPO, CL may suppress group performance by impacting group creativity.

### 5.1 Theoretical implications

Our study is expected to make several contributions to the literature. First, our analysis adds value to the emerging multilevel theories in management practices. As argued by Peccei and van De Voorde (2019), “the field of human resource management (HRM) has lagged behind other areas of management scholarship in this respect, with the dominant paradigm in HRM still being on that is rooted in a single-level perspective typically focusing on the individual or organizational level” (p. 787). In this respect, this research deviates from the conventional scholarly practice of examining management practices at a singular level by studying multilevel mechanisms, thereby extending the existing multilevel paradigm and reaching to the final outcome such as performance in management practices research.

Second, this research investigates the frameworks that elucidate how interactive predictors influence performance via creativity at both individual and group levels. It responds to Mumford’s (2003) observation that current literature on creativity needs to focus more on understanding its effects. Our multilevel results offer evidence and suggest that they may provide a robust framework for comprehending the impacts of creativity. In this vein, the creativity and performance literature can be enriched.

Third, we have developed this paper to explain how CL affects performance via creativity at individual and group levels. Our survey responds to the calls to consider contextual factors and complementarities in understanding the influence of leadership as a critical human resource on creativity, as suggested by Jansen et al. (2009). Specifically, we explored HPWS and GPO’s moderating effects on CL-creativity performance at individual and group levels. This provides research evidence that analyzes the theoretical connections between strategic human resource management (SHRM), leadership streams, and creativity research (Crossan and Apaydin, 2010). We comprehensively understood the relationship between strategic capacity and leadership (Wright et al., 2005) by offering theoretical explanations supported by empirical evidence and examining their impact on creativity. By doing so, we shed light on micro-foundations for organizational capabilities related to creativity and competitive advantages (Helfat and Peteraf, 2015). Our findings offer promising prospects for future research exploring SHRM and leadership.

In conclusion, by linking CL and its boundary conditions, including the HR system and team climate for performance at the individual and group levels, this study explicitly identifies and examines a mechanism that explains how promoting the relationship between CL and creativity can be mutually reinforcing. Connecting these arguments with job demands-resources (JD-R) theory (Demerouti et al., 2001) and broaden-and-build theory (Fredrickson, 2001) at different levels, our study shows that negative or positive work environments would generate or protect resources that contribute to creativity. The results add to the growing evidence for the meaningfulness of leadership and its boundary conditions, leading to organizational performance.

### 5.2 Practical implications

These results have practical implications for individuals and groups seeking to boost creativity and performance within organizational settings. Practitioners often advocate leadership as a universal solution for promoting creativity and performance (Juyumaya and Torres, 2023; Yun, 2023). Our investigation corroborates this assertion up to a point. Charismatic leaders effectively shape their subordinates’ values, work attitudes, beliefs, and other internal psychological characteristics through visionary planning, role modeling, and fulfilling the need for self-actualization (Conger et al., 2000; Sashkin, 1988) to sustain individuals’ vitality and commitment to creative endeavors. In this respect, leaders should prioritize internal training focused on self-development and improvement while leading by example as role models for the group. In contemporary society, organizational development heavily relies on creativity (Hirst et al., 2009, 2011), yet uncertainty is a considerable risk. Within this context, group leaders need to exhibit a spirit of adventure and demonstrate unwavering courage in taking risks as leaders. The more personal risk a leader assumes for the collective cause, the more charismatic they become in the eyes of their followers. Moreover, in case of failure or setbacks encountered along the way, group leaders must possess the fortitude to take responsibility to earn respect from group members.

On the other hand, leaders should integrate their personality traits to cultivate a personal style. As each person possesses unique characteristics, charismatic style cannot be standardized but rather represents an appropriate image based on individual traits such as management and communication styles. By employing diverse approaches and methods, leaders can enable subordinates to perceive their personalized charismatic style, thereby experiencing the leader’s inner strength and vitality. This is conducive to stimulating work enthusiasm and autonomy among group members. Furthermore, the group leaders should strengthen their professional knowledge, skills, and abilities (KSAs) to persuade group members through their KSAs.

More importantly, we have ascertained the interactive mechanisms by which CL, HPWS, and GPO work together in work units. At the individual level, a high level of HPWS may subdue individual creativity motivated by CL, resulting in decreased task performance. Under this circumstance, HPWS may indicate interpersonal norms in the workplace that constrain the link between CL and individual creativity. Conversely, high group-level CL can stimulate higher group creativity within a high level of GPO, leading to improved group performance. Clear performance goals are more conducive to the groups’ focus on performance, thereby motivating creative thinking and problem-solving in overcoming barriers and obstacles (Zhou and George, 2001) within a trust-based environment facilitated by high CL.

Hence, it is crucial for group managers to cautiously manage the HR system and group climate at both individual and group levels. Group leaders should proactively adjust and enhance the HR system while incorporating institutional provisions encouraging individual creativity. Additionally, managers can establish group goals based on specific performance objectives and operational requirements and provide prompt feedback, demonstrate genuine concern for their subordinates, and cultivate a supportive work environment. For example, setting short-term achievable group goals and enabling positive feedback on small wins and accomplishments will boost group members’ confidence and trust, which will be conducive to group creativity and performance improvement.

In sum, effective leadership is pivotal in driving creativity and performance. To optimize the attainment of creative and task goals, managers should leverage the potential of the HR system and group climate to manage individual members and the entire group effectively.

### 5.3 Limitations and directions for future research

This study is subject to several limitations. First, we are required to note some measurement issues. Given the cross-sectional design of this investigation, caution should be exercised when interpreting causality in our findings. Future research can employ a longitudinal study to build and elucidate the causal directions of our predictions.

Second, another noteworthy measurement issue pertains to the self-reported nature of our data collection approach. Although prior research has proved the advantages and superiorities of this method, such as individuals’ ability to better discern variations in their behaviors as responses to specific scenes (Lance et al., 1992), and offer more instances (Parker and Collins, 2010), there exists a potential risk of common method bias associated with self-reporting (Seong et al., 2023). As such, we encourage future scholars to incorporate more objective measures when assessing variables such as creativity and performance. For instance, objective indicators of creativity could include metrics like the number of patents and suggestions (Shin and Zhou, 2007; Zhou and Shalley, 2003).

Third, this paper exclusively focuses on the effects of CL style on creativity and performance. Future studies should also focus on other leadership styles, such as transformational and ethical leadership.

Fourth, our results primarily discuss the mediating role of individual- and group-level creativity in the relationship between multilevel CL and task and group performance. Nevertheless, it is valuable to acknowledge that the mediating mechanisms in the relationships between CL and outcome variables may vary, and further investigation into the “black box” relationship between CL and creativity at both individual and group levels is warranted. Future research can explore additional mediation pathways by including affect variables such as trust and psychological empowerment. Additionally, since CL is not a panacea, it is crucial to appreciate its boundary conditions. We should probe further moderation variables that can either aggrandize or restrict the effects of CL on creativity and performance.

Finally, the companies where we collected data were private sector firms in South Korea. Even though both were located in South Korea, both companies were famous in their field of service and manufacturing. They belong to global firms in the relevant fields. In the future study, we need to expand the sample of survey to the different organizational context including start-ups or public sector firms.

Despite these limitations, our analysis represents an initial attempt to elucidate the multilevel relationships among CL, creativity, and performance while certifying HPWS and GPO’s boundary effects. As a result, our study has extended our understanding regarding the role of leadership in cultivating creativity, raising performance, and making meaningful contributions to management practices literature.

## Data Availability

The raw data supporting the conclusions of this article will be made available by the authors, without undue reservation.
